# Transplantation or rurality? Migration and HIV risk among Chinese men who have sex with men in the urban areas

**DOI:** 10.1002/jia2.25039

**Published:** 2018-01-12

**Authors:** Chuncheng Liu, Rong Fu, Weiming Tang, Bolin Cao, Stephen W Pan, Chongyi Wei, Joseph D Tucker, M. Kumi Smith

**Affiliations:** ^1^ University of North Carolina at Chapel Hill Project‐China Guangzhou China; ^2^ Department of Sociology University of California San Diego CA USA; ^3^ School of Medicine University of North Carolina at Chapel Hill Chapel Hill NC USA; ^4^ Dermatology Hospital Southern Medical University Guangzhou China; ^5^ School of Media and Communication Shenzhen University Shenzhen China; ^6^ Department of Public Health Xi'an Jiaotong‐Liverpool University Suzhou China; ^7^ School of Public Health Rutgers University New Brunswick NJ USA

**Keywords:** men who have sex with men, migration, HIV, China, healthcare access

## Abstract

**Introduction:**

Migration of men who have sex with men (MSM) from rural to urban areas is common across low‐ and middle‐income countries and is widely believed to contribute to elevated HIV risk among migrant MSM in urban areas. Little consensus exists on whether their risk is due to their transplantation or their being from resource‐constrained rural areas. This study seeks to clarify the relationship between migration and HIV risks by comparing differences in HIV‐related risky sexual behaviours and healthcare utilization across competing conceptualizations of migratory statuses.

**Methods:**

In July 2016, MSM ≥16 years old currently residing in one of eight urban cities in China were recruited for an online cross‐sectional survey, which collected information on socio‐demographics, sexual behaviours, HIV care‐seeking behaviours, and healthcare utilization. Based on a question about residency status, each participant was classified as an urban local resident, urban transplant, or rural transplant. Multivariable multinomial logistic regression was used to examine the associations between risky behaviours and healthcare utilization among these three groups.

**Results:**

Among 2007 MSM, the proportion of local, urban transplant and rural transplant were 32% (648/2007), 24% (478/2007), and 44% (881/2007), respectively. Compared with urban local resident MSM, urban transplant MSM were more likely to have ever tested for HIV (adjusted odds ratio (aOR) = 1.39, 95% confidence interval (CI): 1.08 to 1.80). Compared with urban transplant MSM, rural transplant MSM were less likely to have utilized any governmental sexual health services in the past three months (aOR = 0.75, 95% CI: 0.60 to 0.93), ever tested for HIV (aOR = 0.77, 95% CI: 0.61 to 0.96), ever initiated antiretroviral therapy (ART) (aOR = 0.16, 95% CI: 0.05 to 0.52), and ever purchased sex (aOR = 0.57, 95% CI: 0.38 to 0.85). No other significant differences were found in sexual behaviours among three groups.

**Conclusions:**

The widely used local/migrant categorization obscures important differences in HIV risk present between urban/rural subgroups among them. Previous studies of HIV risks in Chinese “migrant” may have failed to consider the role of structural factors such as discrimination or barriers to healthcare when interpreting their findings of higher HIV prevalence in this population. Low ART uptake among rural transplant MSM in this study is particularly concerning and underscore the need for HIV‐related interventions tailored for this group.

## 1 Introduction

The HIV epidemic in men who have sex with men (MSM) is expanding in most countries 1, 2, 3, with steep increases in reports of new HIV diagnoses in several low‐ and middle‐income countries (LMIC) 2, 3, 4, 5. Elevated HIV risk in this population is largely explained by the biological risks associated with anal sex 6, and the large portion of condomless sex taking place in the context of casual partnerships 3. More distal factors such as network effects, social stigma, and migration have also been recognized as key mediators of HIV risk in this population 3, 4.

In many studies on MSM, migration has been particular well examined as an HIV risk factor. Scholars have documented notable trends globally in MSM's migrating from rural area or small cities to larger cities, where the anonymity of city life permits less traditional sexual lifestyles and same sex partners are relatively easier to find 7, 8, 9. Studies of this type of “sexual migration” have reported associations between transplantation and HIV risk in the form of more sexual partners and riskier sexual practices in the big city 11, 12. Structural factors such as social and cultural barriers 8, 10, 11, 13 are also thought to decrease the likelihood of migrant MSM of having stable community support or reliable HIV‐related healthcare access. Some studies particularly emphasized the disadvantage of the migrant MSM with rural origin, who are more likely to internalize the MSM and HIV‐related stigma and reluctant to participate into public health activities due to the more homophobic and sexual health absence grow‐up environment 8, 10, 13, 14, 15.

In China, MSM account for more than one‐third of new HIV cases 16 and the domestic HIV epidemic among MSM is in a rapid expansion phase 3, 17. There is extensive research on MSM and migration and they often find a higher HIV prevalence among migrant MSM compared with its local counterpart 18, 19, 20, 21, 22. However, conclusive evidence of the magnitude or direction of the link between migration and HIV risk and the underlying mechanism remains elusive. While some studies have attributed HIV risk behaviours more common in migrant MSM to them being outsiders (i.e. a transplant) 19, 23, 24, others have attributed their HIV risk behaviours to them being from disadvantaged rural areas 7, 25, 26. Discrepancies in definitions of “migrants” or in sampling design further convolute the relationship between migration and HIV risk. Many studies fail to account for differences in place of origin (urban vs. rural) among migrant MSM 18, 19, 20, 22. While some studies account for the place of origin focused exclusively on the migrant MSM population, and did not compare them to non‐migrant MSM populations 21, 26, 27, 28.

In order to better distinguish the effects of transplant status (local/transplant) versus place of origin status (urban/rural) on HIV risk in so‐called “migrant” MSM in China, we analysed data from an online survey conducted among MSM living in urban areas, in which we compared associations between each type of statuses with common HIV‐related risky sexual behaviour and healthcare utilization. Specifically, we classified participants according to whether they were transplants as well as by their place of origin status (urban/rural status; details below). In so doing, this analysis identifies distinctive sources of HIV risk faced by MSM in the following three categories: urban local resident, urban transplant, and rural transplant. Our findings can both guide future interventions in this population as well as ongoing research on the role of migration in the spread of HIV.

## 2 Methods

### 2.1 Study participants

Study data were collected through an online survey conducted between July and August 2016 in eight urban cities (Guangzhou, Shenzhen, Zhuhai, Jiangmen, Jinan, Qingdao, Jining, and Yantai) from Guangdong and Shandong Provinces, China. Participants were recruited from Blued (Blue Brother, Beijing, China), China's most widely used location‐based gay social networking mobile phone application, comparable with Grindr in the west. Based on users' geolocation, Blued account holders living in one of the eight study cities at the time of study were recruited via a private advertisement message sent from Blued's official account. The private message delivered to users' account mailbox. The message included information on the study background and the terms of survey participation (i.e. risks and benefits), and also provided a link to the survey. Users who clicked on the link were directed to the survey hosted by Sojump Survey Software (Sojump, Shanghai, China).

Eligible participants were born biologically male, were currently living and planning on living in their respective cities for the next 12 months, had oral or anal sex with another man at least once during his lifetime, and were at least 16 years of age. To minimize issues of repeat surveys by the same individuals, we used a screening system in which participants have to verify their phone numbers before taking the survey. A participant must submit the verified code that sent to his phone to enter the survey. Each phone number could only be associated with a single survey submission. Multiple attempts of survey‐taking would be blocked by the system. Eligible survey participants were invited to take part in the survey after signing an online consent form. Participants who completed the survey received 50 Chinese yuan mobile top‐up (about 7 USD) as monetary compensation after finishing the survey.

## 3 Measures

### 3.1 Exposure and outcome

In this study, we classified the participants into three exposure categories: (1) MSM local to the urban area where they lived (“urban local residents”), (2) those who had transplanted from another urban area (“urban transplant”), and (3) those who transplanted from a rural area (“rural transplant”). We used each individuals' reported household registration type, or *hukou*, to measure one's migratory status. The *hukou* refers to China's national household registration system, through which citizens access legal status and social services such as social welfare, public healthcare and education. Every *hukou* contains two pieces of information: location of registered residence (i.e. an address) and classification as urban or rural. The *hukou* status is assigned based on where the resident was born 29, 30. Tight restrictions discourage, but do not prohibit, changing one's *hukou* 30. Therefore, an individual's registered place and urban/rural classification in his/her *hukou* is thought to be the proxy for the environment in which one was born and raised. It has been widely used in the existing HIV literature to classify individuals into “migrants” and non‐migrants 7, 19, 20, 21, 22, 23, 24, 26, 31, 32.

Our definition of a “transplant” as someone who had been living outside of from his registered residence place for more than half of the previous (>6 months) 12 months, was based on definitions of temporary residence defined in the National Bureau of Statistics of China 33. Urban transplants were therefore defined as those holding an urban *hukou* but who had relocated to one of the eight study cities for >6 months within the last year. Rural transplant MSM were defined as MSM with a rural *hukou* who had relocated to one of the eight study cities and had been living there for >6 months in the past year. Given our sample composition, our analysis did not include any MSM living in rural area, restricting our ability to consider the role of rural MSM living in their local setting.

Our outcomes included HIV‐related risk behaviours and healthcare utilization. For the former, the variables included lifetime history of buying or selling sex, any condomless sex and multiple sexual partners in the past three months; for the latter, the variables included the utilization of any government sexual health services in the past three months, ever tested for HIV, and ever initiated antiretroviral therapy (ART) among those who reported being HIV infected. Most HIV‐related sexual health services in China are provided by public disease control agencies. Some community‐based organizations may also provide similar services through programs that mostly supported by government funding 34. Recent utilization of free government sexual health services was defined as having accessed any of the following services in the past three months: free condoms and lubricant, peer led sexual education, HIV and STD screening and treatment, and pamphlets on AIDS/STD related information.

### 3.2 Covariates

Participants reported socio‐demographic information, such as age, education, marital status, annual income, sexual orientation, and status of disclosure of sexual orientation. Education and income levels were categorized according to the conventions of the Chinese national population census, with those reporting a high school education or less classified as “less educated”^35^ and those with annual income of less than 63,920 RMB (about 9200 USD) in urban areas classified as lower income 36. Sexual orientation was defined as those indicating that they identify as “gay” as opposed to “straight,” “bisexual,” “other.” Disclosure of sexual orientation was defined as having ever discussed sexual orientation or same‐sex behaviours with someone other than a sexual partner (e.g. friends or family).

### 3.3 Statistical analysis

**Figure 1:**
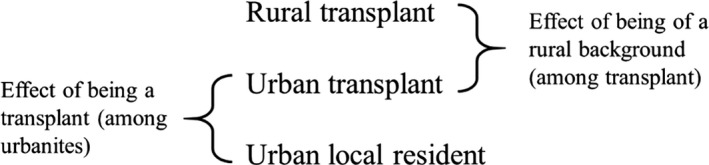
Illustration of the analytic comparisons made to explore the contrasting effects of transplant status and urban/rural status in the 2007 MSM recruited online from eight Chinese cities. MSM, men who have sex with men.

Descriptive analyses compared differences across urban local residents, rural transplants and urban transplants. Statistically significant differences were assessed by examining overlap of 95% confidence intervals (CIs). Multi‐nonnominal logistic regression models were then used to compare odds ratio of reporting HIV‐related risk behaviours and healthcare utilization among the three groups. Odds ratios comparing urban transplants to urban local residents were used to measure the effect of being a transplant on HIV‐related outcomes as it applied to MSM with urban *hukous*. In contrast, odds ratios comparing rural transplants to urban transplants showed the effect of being a rural origin on HIV‐related outcomes as it applied to transplant MSM (Figure 1). Model adjustment minimized confounding by basic socio‐demographic and behavioural variables identified through directed acyclic graphs, an approach used to obtain unconfounded effect estimates when considering multiple potential confounders and which addresses several methodological shortcomings with traditional approaches including multiple hypothesis testing (i.e. inclusion of variables meeting a minimum threshold of statistical significance in univariable models) or automated variable selection methods 37. All analyses were conducted in SAS version 9.4 (Cary, NC, USA) and SPSS version 22.0 (Armonk, NY, USA). In the model, we defined statistical significance as *p*<0.05.

### 3.4 Ethical statement

The study obtained approval from the ethics review committees at the Guangdong Provincial Centre for Skin Diseases and STI Control, the University of North Carolina at Chapel Hill, and the University of California, San Francisco prior to survey launch.

## 4 Results

Among 2007 eligible participants who completed the survey. The proportion of the local, urban transplant and rural transplant was 648 (32%), 478 (24%), and 881 (44%), respectively. The median age of the participants was 25 years old (Range: 16 to 64). A majority of men reported an education level higher than high school (64.4%), were classified as lower income (70.9%), never married (85.5%), and self‐identified as gay (72.1%) (Table 1).

**Table 1:** Socio‐demographic, behavioural and self‐reported HIV‐related characteristics of 2007 MSM recruited online from eight Chinese cities

	Total (N = 2007)	Urban local resident MSM (N = 648)	Urban transplant MSM (N = 478)	Rural transplant MSM (N = 881)
	% (95% CI)	% (95% CI)	% (95% CI)	% (95% CI)
Median Age (SD)	25 (6.45)	26 (7.15)	25 (6.15)	24 (5.95)
Education: higher than high school	64.5 (62.4 to 66.6)	**74.4 (71.0 to 77.7)**	**76.2 (72.3 to 80.0)**	**50.9 (47.6 to 54.2)**
Annual income less than 9200 USD	70.9 (68.9 to 72.9)	**67.6 (64.0 to 71.2)**	**60.5 (56.1 to 64.8)**	**79.0 (76.3 to 81.7)**
Never married	85.5 (83.9 to 87.0)	**80.7 (77.7 to 83.7)**	**90.8 (88.2 to 93.4)**	**86.0 (83.7 to 88.3)**
Self identifies as gay^a^	72.1 (70.1 to 74.1)	71.1 (67.7 to 74.6)	75.5 (71.7 to 79.4)	70.9 (67.9 to 73.9)
Ever disclosed same sex behaviours^b^	67.2 (65.2 to 69.3)	69.4 (65.9 to 73.0)	70.1 (66.0 to 74.2)	64.0 (60.8 to 67.2)
Ever purchased sex	8.2 (7.0 to 9.4)	9.1 (6.9 to 11.3)	10.9 (8.1 to 13.7)	6.0 (4.4 to 7.6)
Ever sold sex	8.6 (7.4 to 9.8)	9.4 (7.2 to 11.7)	9.8 (7.2 to 12.5)	7.4 (5.7 to 9.1)
More than one male sexual partner in the past three months	30.1 (28.1 to 32.1)	30.1 (26.6 to 33.6)	32.4 (28.2 to 36.6)	28.8 (25.8 to 31.8)
Ever had condomless sex with male sexual partner in the past three months	28.1 (26.1 to 30.1)	29.8 (26.3 to 33.3)	28.7 (24.6 to 32.7)	26.6 (23.6 to 29.5)
Utilized any governmental sexual health services in the past three months^c^	61.2 (59.0 to 63.2)	**60.3 (56.6 to 64.1)**	**66.9 (62.7 to 71.2)**	**58.5 (55.2 to 61.7)**
Ever tested for HIV	63.2 (61.1 to 65.3)	**62.8 (59.1 to 66.5)**	**69.6 (65.8 to 74.0)**	**59.8 (56.6 to 63.1)**
* *Among those ever tested (N = 1268) Ever diagnosed with HIV among testers^d^	7.6 (6.1 to 9.0)	7.9 (5.2 to 10.5)	6.9 (4.2 to 9.6)	7.8 (5.5 to 10.1)
* *Among diagnosed HIV positives (N = 96) Ever initiated ART	43.8 (33.8 to 53.7)	**50.0 (32.7 to 67.3)**	**65.2 (45.8 to 84.7)**	**26.8 (13.3 to 40.4)**

Bold format indicates statistically significant differences among three groups, which means no overlap between 95% CI for at least two groups). MSM, men who have sex with men; CI, confidence interval; ART, antiretroviral therapy.

a Sexual orientation was defined as those indicating that they identify as “gay” as opposed to “straight,” “bisexual,” “other.”

b Disclosure to others was defined as sharing same sex behaviours to anyone other than a sexual partner (e.g. friends or family members).

c Governmental sexual health services include free distribution of condoms or lubricant, peer education, STD diagnosis or treatment, and distribution of pamphlets regarding HIV/STI.

d HIV status was self‐reported.

A significantly lower proportion of rural transplants reported having more than a high school education compared with urban local residents and urban transplants (50.9%; 95% CI: 47.6 to 54.2% vs. 74.4%; 95% CI: 71.0 to 77.7 and 76.2%; 95% CI, 72.3 to 80.0, respectively). The same group was also significantly more likely to report a below‐median annual income compared with urban local residents and urban transplants (79.0%; 95% CI: 76.3 to 81.7 vs. 67.6%; 95% CI: 64.0 to 71.2 and 60.5%; 95% CI: 56.1 to 64.8, respectively). In terms of marital status, urban local residents were the most likely to be never married (80.7%; 95% CI: 77.7 to 83.7), though the difference was of borderline significance.

### 4.1 Associations between transplant status and HIV‐related behaviours

In adjusted models comparing transplants from other urban areas with urban local residents (Table 2), MSM who had transplanted were more likely to have ever been tested for HIV (adjusted odds ratios (aOR) = 1.39, 95% CI: 1.08 to 1.80). In crude models, MSM who had transplanted were also more likely to have utilized governmental sexual health services in the past three months (OR = 1.33, 95% CI: 1.04 to 1.70). Detailed item‐based result of governmental sexual health services utilization comparison among three groups could be found in Tables S1, S2, and S3.

**Table 2:** Adjusted odds ratios comparing odds of reporting listed HIV‐related outcomes in urban transplant versus urban local resident MSM (referent), N = 2007

	Urban transplant versus urban local resident MSM	
	OR (95% CI)	aOR (95% CI)
Sexual behaviours		
Ever purchased sex	1.22 (0.82, 1.81)	1.37 (0.92, 2.04)
Ever sold sex	1.05 (0.70, 1.57)	1.15 (0.77, 1.71)
Had condomless sex with male sexual partner in the past three months	0.95 (0.73, 1.23)	1.10 (0.77, 1.32)
More than one male sexual partner in the past three months	1.11 (0.86, 1.43)	1.11 (0.86, 1.45)
Healthcare utilizations		
Utilized any governmental sexual health services in the past three months	**1.33 (1.04, 1.70)**	1.23 (0.97, 1.57)
Ever tested for HIV	**1.37 (1.07, 1.80)**	**1.39 (1.08, 1.80)**
Ever initiated ART (among HIV‐positive individuals, N = 96)	1.88 (0.62, 5.65)	1.57 (0.47, 5.30)

MSM, men who have sex with men; CI, confidence interval; aOR, adjusted odds ratio; ART, antiretroviral therapy. Multivariable model controlled for age, education, income, marital status, disclosure status, and the province of residence. Bold format indicates statistically significant result (*p*<0.05).

### 4.2 Associations between urban/rural status and HIV‐related behaviours

In adjusted models comparing urban and rural transplants (Table 3), MSM of rural backgrounds were significantly less likely to have ever purchased sex (aOR = 0.57, 95% CI: 0.38 to 0.85), to have utilized any governmental sexual health services in the past three months (aOR = 0.75, 95% CI: 0.60 to 0.93) or to have ever tested for HIV (aOR = 0.77, 95% CI: 0.61 to 0.96). Among those self‐reported to be HIV positive (7.5%, 96/1268), rural transplants were far less likely than their urban counterparts to have ever initiated ART (aOR = 0.16, 95% CI: 0.05 to 0.52). Compared with crude models, adjustment for confounding did not significantly alter model outcomes, with the more notable differences observed in the slightly lowered likelihood of rural MSM having ever tested for HIV and the even greater likelihood of those among them with self‐reported HIV to have never initiated ART. Comparison between urban local resident versus rural transplant MSM could be found in Table S4.

**Table 3:** Adjusted odds ratios comparing odds of reporting listed HIV‐related outcomes in rural transplant versus urban transplant MSM (referent), N = 2007

	Rural transplant versus urban transplant MSM	
	OR (95% CI)	aOR (95% CI)
Sexual behaviours		
Ever purchased sex	**0.52 (0.35, 0.78)**	**0.57 (0.38, 0.85)**
Ever sold sex	0.73 (0.49, 1.08)	0.68 (0.46, 1.01)
Had condomless sex with male sexual partner in the past three months	0.90 (0.70, 1.15)	0.94 (0.73, 1.23)
More than one male sexual partner in the past three months	0.84 (0.66, 1.08)	0.98 (0.76, 1.25)
Healthcare utilization		
Utilized any governmental sexual health services in the past three months	**0.71 (0.56, 0.88)**	**0.75 (0.60, 0.93)**
Ever tested for HIV	**0.64 (0.51, 0.81)**	**0.77 (0.61, 0.96)**
Ever initiated ART (among HIV‐positive individuals, N = 96)	**0.20 (0.07, 0.59)**	**0.16 (0.05, 0.52)**

MSM, men who have sex with men; CI, confidence interval; aOR, adjusted odds ratio; ART, antiretroviral therapy. Multivariable model controlled for age, education, income, marital status, disclosure status, and the residence province. Bold format indicates statistically significant result (*p*<0.05).

## 5 Discussion

Our comparison of the associations between a three‐category definition of migrant status and HIV‐related outcomes suggest that the vulnerabilities faced by this group are layered and multifaceted. When compared by their transplant status, for example, urban transplant MSM were found to have higher odds of having ever tested for HIV compared with their local counterpart. When transplant MSM were compared across urban/rural status, however, those of rural backgrounds had a lower odds of reporting risky behaviours and of accessing to the HIV‐related healthcare, relative to their urban counterpart. These findings urge a rethinking of the widely used but loosely defined term, “migrant,” as it is used refer to underlying processes driving the HIV epidemic in Chinese MSM. Although migration likely plays an important role in the spread of HIV in China 18, 19, 20, 21, 22, epidemiological reports on migrant MSM to date cannot fully answer this question due to the lack of more nuanced to understand the drivers of ongoing HIV acquisition. Our findings provide a more realistic assessment of the vulnerabilities faced by migrant MSM living in urban areas, and underscore the need to reexamine past approaches to exploring the effect of migration on HIV risk.

The relative lack of differences in reported sexual behaviours among our study participants of different migration backgrounds departs from existing research on this topic in China 18, 19, 23, 24, 25. Upon closer examination, however, the strength of the evidence on which past conclusions are based vary greatly. For example, a study of MSM in Guangzhou concluded that transplant MSM had riskier sexual behaviours as compared with local MSM 19, despite listing results indicating no statistically significant differences in behaviours across these two groups. Similarly, a second national study concluded that transplant MSM partake in riskier sex than their local counterparts (examples cited include lower condom use or more commercial sex partners), while the finding is based mainly on the inference from the higher HIV prevalence among transplants rather than on explicit questions regarding their behaviours 18. A third study in Wuhan reported riskier sex among MSM who were rural‐to‐urban transplants, a conclusion based on the small subset of participants reporting same sex behaviours (N = 54 or 3.1% of the sample), whose answers regarding frequency of commercial sex or condom use did not specify the sex of the partner 25. A final study on MSM in Jinan used a classification system most comparable to this analysis, which noted significantly higher rates of condomless anal intercourse in transplant MSM 23. The lack of any significant differences in condomless sex and multiple sexual partners leads us to hypothesize that it may be due to contrasting sampling method (respondent driven sampling vs. online recruitment) as well as secular trends in the eight years between when the two studies were conducted.

Compromised HIV testing and other healthcare utilization patterns among rural transplants suggests that attributes specific to their rural backgrounds pose unique challenges when such individuals relocate to a new city. Past findings on health‐seeking behaviours among Chinese migrant MSM have only documented disparities in healthcare utilization across local versus transplants 7, 19, 28, 32. Although many HIV‐related care prevention services for MSM are subsidized by the Chinese government 34, awareness of these services or their accessibility to clients may be particularly low for rural transplant MSM because of their relevantly lower level of education and sexual health literacy 7, 14, 19, 28. Traditional routine interventions on MSM might also face greater barriers in reaching rural transplant MSM who may have more identity struggles or be less likely to take part in health promotion events marketed towards gay men given their experiences of intense stigma directed towards same‐sex behaviours in their rural grow‐up environments 7, 38, 39. In addition, social marginalization 40, discrimination 14, 38, and their effects on mental health and quality of life 41, 42 have been documented in MSM who migrate in other parts of the world and are thought to contribute to risky behaviours and lower self‐efficacy 9, 13. Lastly, the prospect of a possible HIV diagnosis may also be particularly undesirable for rural transplants, given potential threats it may pose to one's housing or job security 14, 33. While for urban transplants, their generally higher socio‐economic status, access to better healthcare resources, and stronger social support network 29, 43, may equip them with better coping mechanisms and self‐efficacy to confront the HIV testing.

The overall rate of ART uptake observed in our sample of MSM living with HIV (43.8%, 42/96) is a far from the UNAIDS goal of 90% of HIV infected persons on treatment 44. This finding highlights an important health service gap in this population, particularly in light of the country's free national ART program which provides free drugs and treatment for opportunistic infections for all people living with HIV. Though the rate of uptake in our sample is slightly lower than those reported by past analyses of ART initiation in China whether in a national cohort (48.9%) 45 or in convenience samples of HIV‐positive MSM (54.3% to 75.2%) 46, 47, 48. However, direct comparisons are challenging given the fact that past studies were restricted to persons already engaged in care or to those under a certain CD4 cell count. The exceptionally low rates of ART uptake rate among rural transplant MSM (26.8%) relative to their urban counterparts MSM is particularly troubling, and suggests that this group faces further barriers relate to their rural background. Accessing HIV‐care in China imposes considerable administrative responsibilities on patients including tasks such as registering with a local disease control centre, preparation of extensive paperwork to prove care eligibility, and undergoing complex enrolment procedures at a registered hospital, all of which can be especially daunting to those with limited social capital or health literacy 25, 29, 47, 49, 50. Meanwhile, Chinese rural transplants more often find work in informal labour markets without job security or health insurance 29, 31, 43, 50, which may contribute to an overall disengagement with medical services, even those freely available such as HIV testing and ART for those infected. Others have also noted that many people from rural area may lack an understanding to fully grasp the severity of HIV disease due to a particular lack of sexual health education in the countryside's basic education 14, 49, 50.

Our study findings have important policy implications. First, urban health authorities should consider ways to improve healthcare utilization, particularly among those from rural areas. Formal mechanisms to solicit feedback from patients, members of the LGBTQ community, and persons of rural background in the city and countryside should be considered as part of intervention design. Community participatory methods such as crowdsourcing may also be helpful for soliciting community feedback 51, 52. Second, our findings underscore the urgent need for interventions to address structural barriers to sexual health services, such as lack of HIV/STD awareness among rural transplant MSM or institutional stigma in healthcare settings, beyond just those seeking to affect individual behaviours of rural transplant community. More intervention activities should be conducted in rural transplant aggregated areas. Health researchers, policy makers, and the media must carefully consider effective means of communicating HIV‐related study results to avoid inaccurate accusation and stigmatization on this population. Third, HIV‐related interventions should contain de‐stigmatization content to encourage more MSM to utilize the sexual health services. Sensitization and ethics trainings for healthcare providers could also have a profound impact on MSM's sexual health services utilization. Moreover, considering about the alarmingly low rate of ART uptake in our study, interventions to improve public awareness regarding the importance of early and constant treatment should be conducted to accelerate treatment uptake.

Given the government focus on mitigating the rapidly growing HIV epidemic among Chinese MSM, more research on the impact of migration can be expected in the future 16, 32. Based on findings from this analysis and on a review of the literature, we extend some modest considerations to guide future design and analysis of research related to migration and HIV risk in MSM. First, investigators might consider collecting and analysing more than one aspect of migration to facilitate more nuanced analysis of the various facets of a migrant's lived experience. For example, urban/rural background, contextual factors of migration such as motivations for migrating, distance from home, and circumstances of migration (moved alone or with friends/family), etc. could provide a clearer understanding of the link between migration and health outcomes. Lastly, studies, particularly qualitative studies on rural transplant MSM are needed to better understand their behaviours and needs to design more targeted interventions.

Findings of this study should be considered in light of several limitations. First, our sample did not allow us to fully distinguish between rural transplant's risk attributable to being of a rural background versus that of being a transplant, since the sample did not include rural MSM residing in the local rural area. Second, our participants were recruited from a mobile APP‐based convenience sample, which may under‐sample older or less educated individuals or people in the stable relationship, thereby restricting the generalizability of our study findings 25, 53. On balance, however, this form of recruitment may also allow researchers greater reach into hidden sub‐groups which cannot be reached by traditional methods, such as MSM who have never disclosed their same sex behaviour to others, or who do not visit frequent venues where traditional study recruitment has taken place 53, 54. Third, our survey questionnaire documented several participants' behaviours as a lifetime exposure, precluding our ability to establish a temporal relationship between these behaviours and the participant's migratory experience. It is therefore possible that our analysis will mistakenly attribute behaviours that took place before the transplant left the countryside to his eventual relocation. Though problematic, we feel the likelihood of commercial same‐sex behaviours taking place in the countryside versus the city are far lower given the relative smaller sex market and lack of anonymity in rural life 8. Fourth, we lacked several key healthcare utilization variables such as insurance status, knowledge about local health facilities, or medical expenditures, precluding out ability to more closely examine potential reasons for compromised access. Last, our analyses pertaining to MSM self‐reported to be living with HIV were not verified by biological outcomes, leading to a possible underestimation of the actual HIV prevalence in the sample.

## 6 Conclusions

Our findings suggest that higher HIV prevalence rates in the so called “migrant MSM” documented by past studies may be due to their structural barriers to accessing HIV prevention interventions rather than from individual risk behaviours. By adopting a more nuanced migratory status as the exposure, our study showed that rural background among transplants is a more salient predictor of the poor healthcare utilization. More tailored interventions regarding healthcare access facilitation and HIV awareness should be conducted among rural transplant MSM. Further research guided by more nuanced measures of migratory status will also help elucidate the intersection between male same sex behaviours, migration, and HIV in China and other LMIC.

## Competing interests

We declare no completing interests.

## Authors' contributions

CW and JT conceived the study. CL, RF, WT, CW, JT and MS contributed to the study design. CL, SP, WT and MS provided statistical support. CL and RF drafted the manuscript with inputs from WT, BC, SP, CW, JT and MS. All authors have seen and approved the final version of the manuscript.

## Funding

This work was supported by National Institutes of Health (National Institute of Allergy and Infectious Diseases (NIAID) 1R01AI114310); University of North Carolina (UNC)‐South China STD Research Training Centre (Fogarty International Centre 1D43TW009532); UNC Center for AIDS Research (NIAID 5P30AI050410); University of California San Francisco Center for AIDS Research (NIAID P30 AI027763); and the Bill & Melinda Gates Foundation to the MeSH Consortium (BMGF‐OPP1120138); National Center for Advancing Translational Sciences (UL1TR001111) at the National Institutes of Health. The listed grant funders played no role in any step of this study.

## Supporting information


**Table S1.** Adjusted odds ratios comparing odds of reporting listed governmental sexual health services utilization in the past three months in urban transplant versus urban local resident MSM (referent), N = 2007
**Table S2.** Adjusted odds ratios comparing odds of reporting listed governmental sexual health services utilization in the past three months in rural transplant versus urban transplant MSM (referent), N = 2007
**Table S3.** Adjusted odds ratios comparing odds of reporting listed governmental sexual health services utilization in the past three months in urban local resident versus rural transplant MSM (referent), N = 2007
**Table S4.** Adjusted odds ratios comparing odds of reporting listed HIV related outcomes in urban local resident versus rural transplant resident MSM (referent), N = 2007

## References

[1] HIV/AIDS JUNPo . Global report: UNAIDS report on the global AIDS epidemic 2013. Geneva: UNAIDS; 2013. According to the UNAIDS'estimate the number of new infections in the region increased from. 2015; 21: 22000–47.

[2] vanGriensven F , vande Lind Wijngaarden JW , Baral S , Grulich A . The global epidemic of HIV infection among men who have sex with men. Curr Opin HIV AIDS. 2009;4(4):300–7. 19532068 10.1097/COH.0b013e32832c3bb3

[3] Beyrer C , Baral SD , van Griensven F , Goodreau SM , Chariyalertsak S , Wirtz AL , et al. Global epidemiology of HIV infection in men who have sex with men. Lancet. 2012;380(9839):367–77. 22819660 10.1016/S0140-6736(12)60821-6 PMC3805037

[4] Beyrer C , Sullivan P , Sanchez J , Baral SD , Collins C , Wirtz AL , et al. The increase in global HIV epidemics in MSM. AIDS. 2013;27(17):2665–78. 23842129 10.1097/01.aids.0000432449.30239.fe

[5] Beyrer C , Baral SD , Walker D , Wirtz AL , Johns B , Sifakis F . The expanding epidemics of HIV type 1 among men who have sex with men in low‐ and middle‐income countries: diversity and consistency. Epidemiol Rev. 2010;32(1):137–51. 20573756 10.1093/epirev/mxq011

[6] Seage GR , Mayer KH , Horsburgh CR . Risk of human immunodeficiency virus infection from unprotected receptive anal intercourse increases with decline in immunologic status of infected partners. Am J Epidemiol. 1993;137(8):899–908. 8484381 10.1093/oxfordjournals.aje.a116751

[7] Hu J , Gu X , Tao X , Qian Y , Babu GR , Wang G , et al. Prevalence and trends of HIV, syphilis, and HCV in migrant and resident men who have sex with men in Shandong, China: results from a serial cross‐sectional study. PLoS One. 2017;12(1):e0170443. 28103295 10.1371/journal.pone.0170443 PMC5245858

[8] Lewis NM . Placing HIV beyond the metropolis: risks, mobilities, and health promotion among gay men in the Halifax, Nova Scotia region. Can Geogr. 2015;59(2):126–35.

[9] Bruce D , Harper GW ; Adolescent Medicine Trials Network for HIVAI . Operating without a safety net: gay male adolescents and emerging adults' experiences of marginalization and migration, and implications for theory of syndemic production of health disparities. Health Educ Behav. 2011;38(4):367–78. 21398621 10.1177/1090198110375911 PMC3149744

[10] Wirtz AL , Zelaya CE , Peryshkina A , Latkin C , Mogilnyi V , Galai N , et al. Social and structural risks for HIV among migrant and immigrant men who have sex with men in Moscow, Russia: implications for prevention. AIDS Care. 2014;26(3):387–95. 23875610 10.1080/09540121.2013.819407 PMC3947262

[11] Annes A , Redlin M . Coming out and coming back: rural gay migration and the city. J Rural Stud. 2012;28(1):56–68.

[12] Kelly BC , Muñoz‐Laboy MA . Sexual place, spatial change, and the social reorganization of sexual culture. J Sex Res. 2005;42(4):359–66. 19827241 10.1080/00224490509552292

[13] Lewis NM . Rupture, resilience, and risk: relationships between mental health and migration among gay‐identified men in North America. Health Place. 2014;27 Suppl C:212–9. 24662530 10.1016/j.healthplace.2014.03.002

[14] Saether ST , Chawphrae U , Zaw MM , Keizer C , Wolffers I . Migrants' access to antiretroviral therapy in Thailand. Tropical Med Int Health. 2007;12(8):999–1008. 10.1111/j.1365-3156.2007.01879.x 17697095

[15] Kubicek K , Beyer WJ , Weiss G , Iverson E , Kipke MD . In the dark: young men's stories of sexual initiation in the absence of relevant sexual health information. Health Educ Behav. 2010;37(2):243–63. 19574587 10.1177/1090198109339993 PMC2866104

[16] Guo Y , Li X , Stanton B . HIV‐related behavioral studies of men who have sex with men in China: a systematic review and recommendations for future research. AIDS Behav. 2011;15(3):521–34. 21053064 10.1007/s10461-010-9808-7 PMC8182773

[17] China A . Response progress report. UNAIDS: Ministry of Health of the People's Republic of China; 2012.

[18] Wu Z , Xu J , Liu E , Mao Y , Xiao Y , Sun X , et al. HIV and syphilis prevalence among men who have sex with men: a cross‐sectional survey of 61 cities in China. Clin Infect Dis. 2013;57(2):298–309. 23580732 10.1093/cid/cit210 PMC3689345

[19] Wu J , Wu H , Li P , Lu C . HIV/STIs risks between migrant MSM and local MSM: a cross‐sectional comparison study in China. PeerJ. 2016;4:e2169. 27478695 10.7717/peerj.2169 PMC4950534

[20] Wang B , Li X , Stanton B , Liu Y , Jiang S . Socio‐demographic and behavioral correlates for HIV and syphilis infections among migrant men who have sex with men in Beijing, China. AIDS Care. 2013;25(2):249–57. 22788859 10.1080/09540121.2012.701714 PMC3563352

[21] Mao H , Ma W , Lu H , Wang L , Zheng H , Zhu Y , et al. High incidence of HIV and syphilis among migrant men who have sex with men in Beijing, China: a prospective cohort study. BMJ Open. 2014;4(9):e005351. 10.1136/bmjopen-2014-005351 PMC4166416 25227626

[22] Yu Y , Xu J , Hu Q , Yan H‐J , Wang Z , Lu L , et al. High‐risk behaviour and HIV infection risk among non‐local men who have sex with men with less than a single year's residence in urban centres: a multicentre cross‐sectional study from China. Sex Transm Infect. 2016. 10.1136/sextrans-2016-052744 29348258

[23] Ruan S , Yang H , Zhu Y , Ma Y , Li J , Zhao J , et al. HIV prevalence and correlates of unprotected anal intercourse among men who have sex with men, Jinan, China. AIDS Behav. 2008;12(3):469–75. 18259850 10.1007/s10461-008-9361-9

[24] Liu Y , Li X , Zhang L , Li S , Jiang S , Stanton B . Correlates of consistent condom use among young migrant men who have sex with men (MSM) in Beijing, China. Eur J Contracept Reprod Health Care. 2012;17(3):219–28. 22559259 10.3109/13625187.2012.662544 PMC8182772

[25] Chen X , Yu B , Zhou D , Zhou W , Gong J , Li S , et al. A comparison of the number of men who have sex with men among rural‐to‐urban migrants with non‐migrant rural and urban residents in Wuhan, China: a GIS/GPS‐assisted random sample survey study. PLoS One. 2015;10(8):e0134712. 26241900 10.1371/journal.pone.0134712 PMC4524597

[26] Guo Y , Li X , Song Y , Liu Y . Bisexual behavior among Chinese young migrant men who have sex with men: implications for HIV prevention and intervention. AIDS Care. 2012;24(4):451–8. 22085021 10.1080/09540121.2011.613914 PMC8185875

[27] Guo Y , Li X , Liu Y , Jiang S , Tu X . Disclosure of same‐sex behavior by young Chinese migrant men: context and correlates. Psychol Health Med. 2014;19(2):190–200. 23654216 10.1080/13548506.2013.793367

[28] Song Y , Li X , Zhang L , Fang X , Lin X , Liu Y , et al. HIV‐testing behavior among young migrant men who have sex with men (MSM) in Beijing, China. AIDS Care. 2011;23(2):179–86. 21259130 10.1080/09540121.2010.487088 PMC3076143

[29] Chen J . Internal migration and health: re‐examining the healthy migrant phenomenon in China. Soc Sci Med. 2011;72(8):1294–301. 21435765 10.1016/j.socscimed.2011.02.016

[30] Wei H , Sheng G . China's household registration system reform: process, barriers, and strategies. Rev Econ Res. 2015;03:6–17.

[31] Mi G , Ma B , Kleinman N , Li Z , Fuller S , Bulterys M , et al. Hidden and mobile: a web‐based study of migration patterns of men who have sex with men in China. Clin Infect Dis. 2016;62(11):1443–7. 27129466 10.1093/cid/ciw167 PMC5991978

[32] Zhang L , Xiao Y , Lu R , Wu G , Ding X , H‐z Qian , et al. Predictors of HIV testing among men who have sex with men in a large Chinese city. Sex Transm Dis. 2013;40(3):235. 23403605 10.1097/OLQ.0b013e31827ca6b9 PMC3725775

[33] National Bureau of Statistics of China . The result of population census of China 2016. [Accessed 2016 Dec 11]. Available from: http://www.stats.gov.cn/english/Statisticaldata/CensusData/

[34] Wu Z , Sullivan SG , Wang Y , Rotheram‐Borus MJ , Detels R . Evolution of China's response to HIV/AIDS. Lancet. 2007;369(9562):679–90. 17321313 10.1016/S0140-6736(07)60315-8 PMC7137740

[35] Cai H . Report on China labor‐force dynamic survey. Beijing: Social Sciences Academic Press; 2015.

[36] National Bureau of Statistics of China . Average annual income of China in 2015. 2015 [Accessed 2016 Dec 11] Available from: http://www.stats.gov.cn/tjsj/zxfb/201605/t20160513_1356091.html

[37] Greenland S , Pearl J , Robins JM . Causal diagrams for epidemiologic research. Epidemiology. 1999;10:37–48. 9888278

[38] Li X , Stanton B , Fang X , Lin D . Social stigma and mental health among rural‐to‐urban migrants in China: a conceptual framework and future research needs. World Health Popul. 2006;8(3):14–31. 18277106 10.12927/whp.2006.18282 PMC2249560

[39] Chow EP , Gao L , Koo FK , Chen L , Fu X , Jing J , et al. Qualitative exploration of HIV‐related sexual behaviours and multiple partnerships among Chinese men who have sex with men living in a rural area of Yunnan Province, China. Sexual Health. 2013;10(6):533–40. 24119339 10.1071/SH13097

[40] Wong K , Fu D , Li CY , Song HX . Rural migrant workers in urban China: living a marginalised life. Int J Soc Welf. 2007;16(1):32–40.

[41] Wang B , Li X , Stanton B , Fang X . The influence of social stigma and discriminatory experience on psychological distress and quality of life among rural‐to‐urban migrants in China. Soc Sci Med. 2010;71(1):84–92. 20403653 10.1016/j.socscimed.2010.03.021

[42] Lin D , Li X , Wang B , Hong Y , Fang X , Qin X , et al. Discrimination, perceived social inequity, and mental health among rural‐to‐urban migrants in China. Community Ment Health J. 2011;47(2):171–80. 20033772 10.1007/s10597-009-9278-4 PMC2891847

[43] Li J , Gu Y . Hukou‐based stratification in China's urban labor market. Sociol Stud. 2011;2:48–77.

[44] UNAIDS . 90‐90‐90: an ambitious treatment target to help end the AIDS epidemic. Geneva: Joint United Nations Programme on HIV/AIDS (UNAIDS); 2014.

[45] Zhang F , Dou Z , Ma Y , Zhang Y , Zhao Y , Zhao D , et al. Effect of earlier initiation of antiretroviral treatment and increased treatment coverage on HIV‐related mortality in China: a national observational cohort study. Lancet Infect Dis. 2011;11(7):516–24. 21600849 10.1016/S1473-3099(11)70097-4

[46] Wang M , Zhang H , Wang J , Yu M , Song D , Cao Z , et al. Status and correlates of standardized follow‐up CD4 counting and antiretroviral therapy among HIV‐positive men who have sex with men. Chin J Dis Control Prev. 2012;16(12):1011–4.

[47] Hu X , Chen F , Ding F , Lin X , Wang X , He H , et al. Coverage of HIV related follow‐up intervention and antiretroviral treatment and its correlation among HIV‐positive men who have sex with men of 3 cities in China. Chin J Prev Med. 2015;49(11):945–9. 26833002

[48] Zhou W , Zhao M , Wang X , Schilling RF , Zhou S , Qiu HY , et al. Treatment adherence and health outcomes in MSM with HIV/AIDS: patients enrolled in “one‐stop” and standard care clinics in Wuhan China. PLoS One. 2014;9(12):e113736. 25438039 10.1371/journal.pone.0113736 PMC4249979

[49] Zhang L , Chow EP , Jahn HJ , Kraemer A , Wilson DP . High HIV prevalence and risk of infection among rural‐to‐urban migrants in various migration stages in China: a systematic review and meta‐analysis. Sex Transm Dis. 2013;40(2):136–47. 23321993 10.1097/OLQ.0b013e318281134f

[50] Li X , Fang X , Lin D , Mao R , Wang J , Cottrell L , et al. HIV/STD risk behaviors and perceptions among rural‐to‐urban migrants in China. AIDS Educ Prev. 2004;16(6):538–56. 15585430 10.1521/aeap.16.6.538.53787 PMC1791014

[51] Zhang Y , Kim JA , Liu F , Tso LS , Tang W , Wei C , et al. Creative contributory contests to spur innovation in sexual health: 2 cases and a guide for implementation. Sex Transm Dis. 2015;42(11):625. 26462186 10.1097/OLQ.0000000000000349 PMC4610177

[52] Zhang TP , Liu C , Han L , Tang W , Mao J , Wong T , et al. Community engagement in sexual health and uptake of HIV testing and syphilis testing among MSM in China: a cross‐sectional online survey. J Int AIDS Soc. 2017;20(1):21372 https://doi.org/10.7448/IAS.20.01.21372 28406270 10.7448/IAS.20.01/21372 PMC5515028

[53] Ross MW , Tikkanen R , Månsson S‐A . Differences between Internet samples and conventional samples of men who have sex with men: implications for research and HIV interventions. Soc Sci Med. 2000;51(5):749–58. 10975234 10.1016/s0277-9536(99)00493-1

[54] Guo Y , Li X , Fang X , Lin X , Song Y , Jiang S , et al. A comparison of four sampling methods among men having sex with men in China: implications for HIV/STD surveillance and prevention. AIDS Care. 2011;23(11):1400–9. 21711162 10.1080/09540121.2011.565029 PMC3202036

