# Supplementary material for: Transplantation or rurality? Migration and HIV risk among Chinese men who have sex with men in the urban areas
Source: J Int AIDS Soc. 2018 Jan 12;21(1):e25039. doi: 10.1002/jia2.25039 (PMC5810344; doi:10.1002/jia2.25039)
Supplement: Supplementary file 1 — Table S1. Adjusted odds ratios comparing odds of reporting listed governmental sexual health services utilization in the past three months in urban transplant versus urban local resident MSM (referent), N = 2007 Table S2. Adjusted odds ratios comparing odds of reporting listed governmental sexual health services utilization in the past three months in rural transplant versus urban transplant MSM (referent), N = 2007 Table S3. Adjusted odds ratios comparing odds of reporting listed governmental sexual health services utilization in the past three months in urban local resident versus rural transplant MSM (referent), N = 2007 Table S4. Adjusted odds ratios comparing odds of reporting listed HIV related outcomes in urban local resident versus rural transplant resident MSM (referent), N = 2007 [file JIA2-21-e25039-s001.docx]

**Additional file 1: Supplementary Tables**

**Supplementary Table 1. Adjusted odds ratios comparing odds of reporting listed governmental sexual health services utilization in the past three months in urban transplant versus urban local resident MSM (referent), N=2007**

|  | Urban transplant versus urban local resident MSM | |
| --- | --- | --- |
|  | OR (95% CI) | aOR (95% CI) |
| Free condoms | **1.34 (1.07, 1.68)** | **1.32 (1.03, 1.69)** |
| Free lubricant | **1.31 (1.00, 1.71)** | **1.41 (1.06, 1.91)** |
| Peed led sexual education | 1.31 (0.96, 1.77) | 1.17 (0.84, 1.62) |
| HIV and STD screening and treatment | **1.32 (1.04, 1.68)** | 1.14 (0.88, 1.48) |
| Pamphlets on HIV/STD related information | 1.15 (0.89, 1.49) | 1.06 (0.81, 1.40) |

Note: Multivariable model controlled for age, education, income, marital status, disclosure status, and the province of residence. Bold format indicates statistically significant result (p<0.05).

**Supplementary Table 2. Adjusted odds ratios comparing odds of reporting listed governmental sexual health services utilization in the past three months in rural transplant versus urban transplant MSM (referent), N=2007**

|  | Rural transplant versus urban transplant MSM | |
| --- | --- | --- |
|  | OR (95% CI) | aOR (95% CI) |
| Free condoms | **0.75 (0.60, 0.94)** | **0.78 (0.62, 0.99)** |
| Free lubricant | **0.76 (0.58, 0.99)** | 0.79 (0.60, 1.05) |
| Peed led sexual education | 0.77 (0.56, 1.04) | 0.84 (0.61, 1.16) |
| HIV and STD screening and treatment | 0.93 (0.66, 1.32) | 0.82 (0.64, 1.06) |
| Pamphlets on HIV/STD related information | 0.87 (0.67, 1.12) | 0.89 (0.68, 1.16) |

Note: Multivariable model controlled for age, education, income, marital status, disclosure status, and the province of residence. Bold format indicates statistically significant result (p<0.05).

**Supplementary Table 3. Adjusted odds ratios comparing odds of reporting listed governmental sexual health services utilization in the past three months in urban local resident versus rural transplant MSM (referent), N=2007**

|  | Urban local resident versus rural transplant MSM | |
| --- | --- | --- |
|  | OR (95% CI) | aOR (95% CI) |
| Free condoms | 1.02 (0.82, 1.26) | 0.97 (0.78, 1.21) |
| Free lubricant | 0.95 (0.73, 1.23) | 0.89 (0.68, 1.17) |
| Peed led sexual education | 1.10 (0.82, 1.46) | 1.01 (0.75, 1.37) |
| HIV and STD screening and treatment | 1.09 (0.87, 1.36) | 1.06 (0.84, 1.34) |
| Pamphlets on HIV/STD related information | 1.05 (0.83, 1.33) | 1.06 (0.83, 1.36) |

Note: Multivariable model controlled for age, education, income, marital status, disclosure status, and the province of residence. Bold format indicates statistically significant result (p<0.05).

**Supplementary Table 4. Adjusted odds ratios comparing odds of reporting listed HIV related outcomes in urban local resident versus rural transplant resident MSM (referent), N=2007**

|  | Urban local resident versus rural transplant MSM | |
| --- | --- | --- |
|  | OR (95%CI) | aOR (95% CI) |
| **Sexual Behaviors** |  |  |
| Ever purchased sex | **1.57 (1.06, 2.30)** | 1.28 (0.86, 1.91) |
| Ever sold sex | 1.31 (0.91, 1.88) | 1.29 (0.89, 1.90) |
| Had condomless sex with male sexual partner in the past three months | 1.17 (0.94, 1.47) | 1.05 (0.83, 1.33) |
| More than one male sexual partner in the past three months | 1.06 (0.85, 1.33) | 0.91 (0.72, 1.15) |
|  |  |  |
| **Healthcare Utilizations** |  |  |
| Utilized any governmental sexual health services in the past three months | 1.09 (0.89, 13.4) | 1.23 (0.83, 1.27) |
| Ever tested for HIV | 1.13 (0.92, 1.40) | 0.87 (0.70, 1.09) |
| Ever initiated ART (*among HIV-positive individuals, N=96*) | **2.73 (1.03, 7.26)** | **3.89 (1.23, 12.32)** |

Note: Multivariable model controlled for age, education, income, marital status, disclosure status, and the province of residence. Bold format indicates statistically significant result (p<0.05).
